# Aquaporin-4 in glioblastoma: a nexus of glymphatic dysfunction, edema, immune evasion, and treatment resistance

**DOI:** 10.3389/fncel.2025.1685491

**Published:** 2025-11-12

**Authors:** Leandro Castañeyra-Ruiz, Ibrahim González-Marrero, Luis H. García-Abad, Emilio Gonzalez-Arnay, Marcial Camacho, Emilia Ma Carmona-Calero, Seunghyun Lee, Celine Thao-Quyen Tran, Brian W. Hanak, Michael Muhonen, Agustín Castañeyra-Perdomo

**Affiliations:** 1 Children’s Hospital of Orange County Research Institute, Orange, CA, United States; 2 Unidad de Anatomía, Departamento de Ciencias Médicas Básicas, Facultad de Medicina, Universidad de La Laguna, San Cristóbal de La Laguna, Spain; 3 Unidad de Farmacología, Departamento de Medicina Física y Farmacología, Facultad de Medicina Universidad de La Laguna, San Cristóbal de La Laguna, Spain; 4 Instituto de Investigación y Ciencias de Puerto del Rosario, Puerto del Rosario, Spain; 5 Department of Neurological Surgery, University of California Irvine, Orange, CA, United States

**Keywords:** AQP4, glioblastoma, glioma, glymphatic system, astrocyte

## Abstract

Glioblastoma (GBM) progression is linked to aquaporin-4 (AQP4), whose functions extend beyond water transport to influence perivascular architecture, immune modulation, edema, and treatment response. In the healthy brain, AQP4 is highly polarized at astrocytic endfeet, supporting perivascular fluid exchange and glymphatic clearance. In GBM, AQP4 is frequently upregulated and mislocalized, correlating with blood–brain barrier (BBB) disruption, impaired directional fluid movement, and peritumoral edema. Peritumoral astrocytic mislocalization of AQP4, together with tumor mass effect, compromises glymphatic function by distorting perivascular spaces and compressing cerebrospinal fluid (CSF)-Interstitial fluid (ISF) exchange zones. We review evidence that AQP4 isoforms (M1 vs. M23) differentially shape motility and membrane organization, and we outline how AQP4-linked signaling axes (e.g., indoleamine 2,3-dioxygenase 1 (IDO1)/tryptophan 2,3-dioxygenase (TDO)-kynurenine–aryl hydrocarbon receptor (AhR) can bias pro-invasive states and immunosuppressive niches enriched with M2-like macrophages). We integrate a four-zone perivascular framework to localize where GBM most perturbs periarterial and perivenous pathways, as well as meningeal lymphatic outflow. Finally, we discuss therapeutic directions spanning AQP4 modulation, isoform balance, and BBB-bypassing delivery strategies. Overall, AQP4 emerges as a mechanistic hub connecting BBB instability, glymphatic impairment, edema, immune evasion, and invasion in GBM.

## Introduction

The Glymphatic system (GS) is a brain-wide fluid clearance network responsible for the exchange of CSF and ISF. It facilitates the removal of metabolic waste and contributes to fluid homeostasis and solute trafficking within the central nervous system (CNS) (Xu et al., 2022). The GS comprises a multi-segmented anatomical continuum, including periarterial influx pathways, CSF–ISF exchange zones, and perivenous efflux routes that ultimately drain into the meningeal lymphatic vessels (MLVs) (Xu et al., 2022; Klostranec et al., 2021; Jessen et al., 2015; Hablitz and Nedergaard, 2021). Central to the function of this system is aquaporin-4 (AQP4), a water channel protein abundantly expressed at astrocytic endfeet, which enables directional fluid movement along perivascular routes (Plog and Nedergaard, 2018; Gomolka et al., 2023).

**Figure 1:**
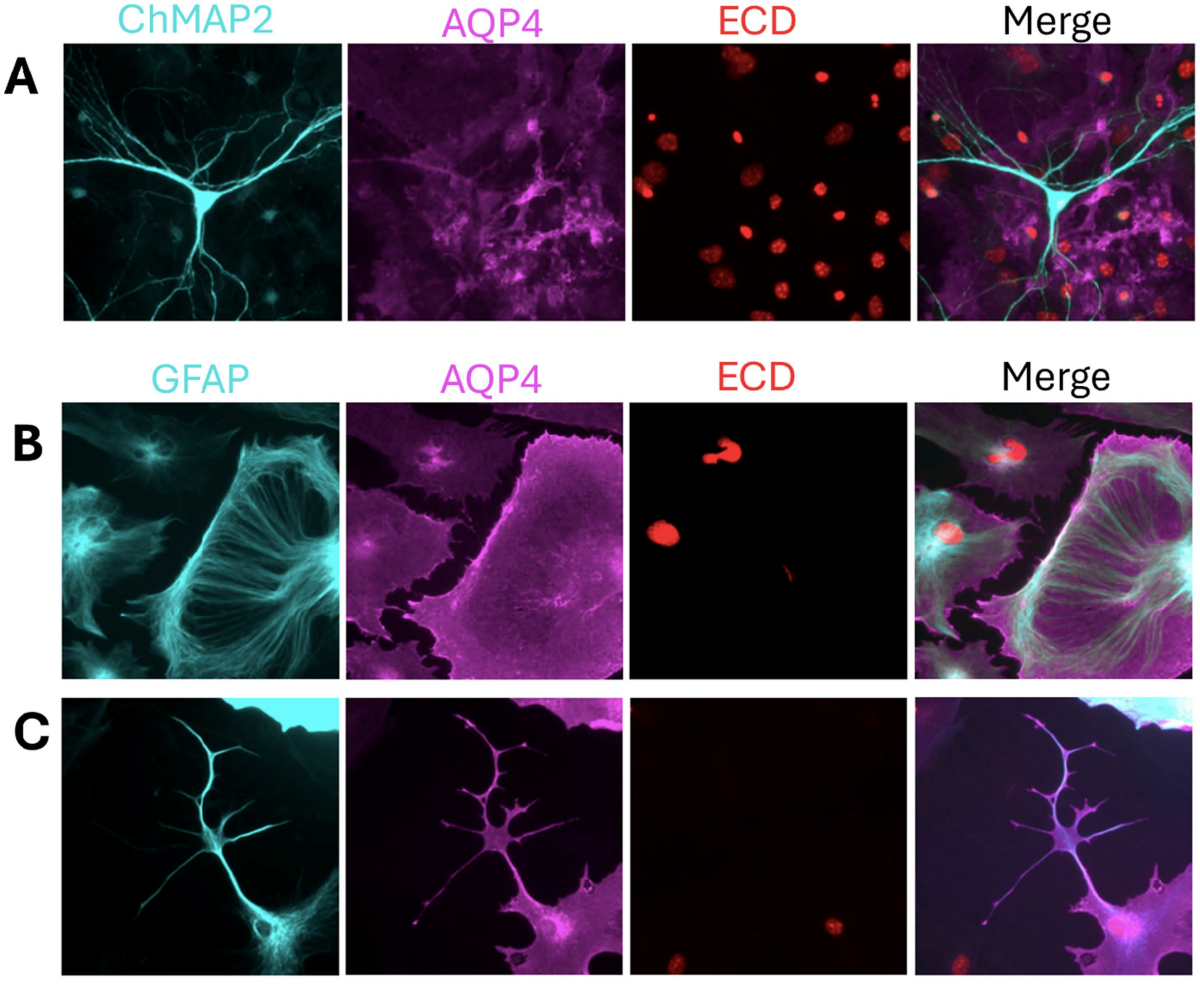
AQP4 glia lineage marker and indicator of morphological diversity. **(A)** Mixed neuron–astrocyte cultures stained with ChMAP2 (neurons, cyan), AQP4 (magenta), and ECD (nuclei, red) show AQP4 expression restricted to astrocytes, confirming its cell-type specificity. **(B,C)** Astrocyte monocultures stained with GFAP (cyan), AQP4, and ECD reveal distinct morphologies. **(B)** Cells with broad lamellipodia and radial GFAP arrays reflect a migratory phenotype, potentially linked to M1-AQP4. **(C)** Stellate astrocytes with membrane-enriched AQP4 suggest a stabilized phenotype, consistent with M23-AQP4 and OAP formation. Samples were fixed with 4% PFA, permeabilized with 0.1% Tween-20, and stained using anti-mouse GFAP (rhodamine red) and anti-rabbit AQP4 (alexa fluor 488). Unpublished image from our laboratory.

Aquaporins have increasingly been associated with the pathophysiology of several conditions, including hydrocephalus and systematic hypertension, since these pathologies have characteristic nervous system water transport alterations (Castaneyra-Ruiz et al., 2016a; Gonzalez-Marrero et al., 2022; Castaneyra-Ruiz et al., 2013; Castaneyra-Ruiz et al., 2016b; Castaneyra-Ruiz et al., 2019; Carmona-Calero et al., 2015); however, beyond its canonical role, specially AQP4 has emerged as an astrocytic lineage marker (Castaneyra-Ruiz et al., 2022a; Castaneyra-Ruiz et al., 2022b; Holst et al., 2019) and a mediator of diverse astrocytic functions, including cellular migration, cytoskeletal remodeling and microenvironmental interactions. These functions are often associated with the dominant AQP4-isoforms expressed in an astrocyte (Pisani et al., 2021; Smith et al., 2014) (Figure 1). These non-canonical roles of AQP4 have generated increasing interest in its contribution to neuropathological states, particularly brain tumors of glial origin, including gliomas and astrocytomas.

Glioblastoma multiforme (GBM) is the most common and lethal form of primary brain tumor in adults, characterized by diffuse infiltration, genetic heterogeneity, and limited responsiveness to current therapies (Batash et al., 2017; Ghosh et al., 2018). Despite multimodal treatment involving surgery, radiation, and chemotherapy, the median survival remains dismal—approximately 15 months (Alifieris and Trafalis, 2015; Wu et al., 2021; Pearson et al., 2020). This poor prognosis is largely attributed to the tumor’s aggressive invasion into surrounding brain parenchyma, its ability to resist conventional therapies, and its manipulation of the immune and fluid microenvironments (van Linde et al., 2017; Yalamarty et al., 2023).

Importantly, AQP4 has been implicated in several aspects of GBM progression, including enhanced cell migration, resistance to apoptosis, disruption of the blood–brain barrier (BBB), and the polarization of tumor-associated macrophages (TAMs) toward an immunosuppressive phenotype (Amiry-Moghaddam, 2019; Simone et al., 2019; Lan et al., 2022). These functions associate AQP4 with tumor-promoting functions.

This review provides a comprehensive synthesis of current knowledge linking glymphatic anatomy with GBM pathophysiology and AQP4 dysfunction. We propose a refined anatomical model of the glymphatic system, including four functional zones, and evaluate the isoform-specific contributions of AQP4 to glioma cell behavior. By integrating molecular, anatomical, and clinical insights, we aim to define the AQP4–glymphatic axis as a critical node in GBM progression and a potential target for future diagnostic and therapeutic strategies.

## Anatomical organization of the glymphatic system

The GS facilitates directional CSF influx, ISF exchange, and metabolic waste clearance from the brain parenchyma (Xu et al., 2022; Iliff et al., 2012). Traditionally divided into three segments—periarterial influx (S1), CSF–ISF exchange (S2), and perivenous efflux (S3) (Xu et al., 2022). Here, we propose a fourth anatomical segment (S4), corresponding to the meningeal perivenous drainage compartment, which is structurally and functionally distinct from its parenchymal counterpart, as described below.

## S1: Periarterial influx region

**Figure 2:**
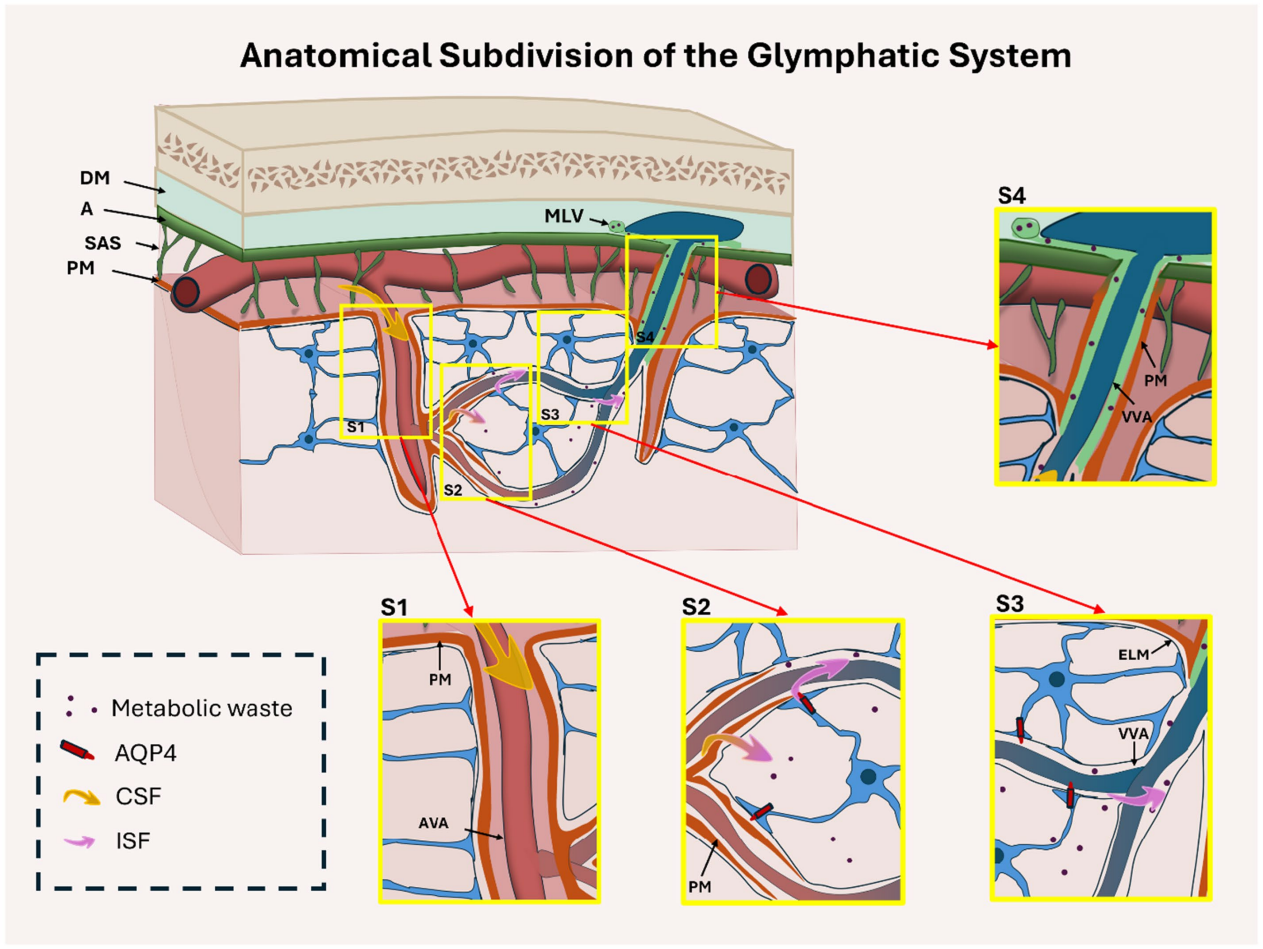
Anatomical organization of the glymphatic system, subdivided into four functional regions: periarterial influx (S1), CSF–interstitial fluid (ISF) exchange (S2), perivenous parenchymal efflux (S3), and meningeal efflux (S4). This cross-sectional diagram shows the brain cortex, subarachnoid space (SAS), and meninges (dura mater [DM], arachnoid [A], pia mater [PM]). In S1, CSF (yellow arrows) enters periarterial spaces through the Virchow-Robin space, located between the arterial vascular adventitia (AVA) and the PM. In S2, the PM becomes discontinuous, enabling CSF–ISF exchange mediated by astrocytic AQP4 channels. In S3, glymphatic fluid flows along perivenous spaces between the external limiting membrane (ELM) and venous vascular adventitia (VVA) within the parenchyma. In S4, flow continues in the meningeal compartment between the VVA and PM toward meningeal lymphatic vessels (MLV). AQP4 is relevant only in the parenchymal segments (S2–S3), but not in S1 or S4. Insets S1–S4 highlight key anatomical features of each region.

This region surrounds arteries that penetrate the brain from the subarachnoid space. The Virchow–Robin space (VRS), located between the arterial vascular adventitia (AVA) and the pia mater (PM), forms the main conduit for CSF entry into the brain interstitium (Hablitz and Nedergaard, 2021). This segment establishes the foundation of glymphatic flow (Figure 2).

## S2: CSF–ISF exchange zone

Here, the pia mater thins and gradually disappears, allowing for the direct interaction of CSF with the interstitial compartment. In this section, AQP4, highly expressed at the astrocytic endfeet, facilitates bidirectional water transport between CSF and ISF, making this region central to solute exchange and fluid balance (Gomolka et al., 2023; Giannetto et al., 2024) (Figure 2).

## S3: Perivenous efflux region (parenchymal)

As glymphatic flow transitions to venous outflow, CSF–ISF passes along perivenous spaces bordered by the external limiting membrane (ELM) and venous vascular adventitia (VVA). AQP4 expression persists in this segment, enabling waste clearance toward the brain surface (Rasmussen et al., 2018; Iliff et al., 2015). Structural disruption of this segment is frequently observed in pathological conditions, including GBM (Figure 2).

## S4: Perivenous efflux region (meningeal)

Beyond the parenchyma, fluid continues along subarachnoid perivenous spaces bordered by the VVA and PM, eventually draining into the meningeal lymphatic vessels (MLVs). AQP4 is not functionally involved in this segment (Figure 2). However, anatomical alterations in this zone—such as compression or obstruction by tumor masses—may still indirectly influence glymphatic function.

This four-zone anatomical model offers a refined framework for investigating region-specific vulnerabilities in glymphatic transport, particularly in pathologies such as GBM, in which edema and stasis predominantly arise in CSF–ISF exchange (S2) and parenchymal perivenous efflux (S3), whereas diminished out flow affects meningeal lymphatic regions (S4) (Ma et al., 2019).

## AQP4 localization and blood–brain barrier integrity

AQP4 not only facilitates glymphatic transport but is also essential for maintaining the structural and functional integrity of the blood–brain barrier (BBB). Under physiological conditions, AQP4 is highly polarized at perivascular astrocytic endfeet, where it mediates bidirectional water flux and contributes to homeostatic exchange between vascular and parenchymal compartments (Warth et al., 2007; Warth et al., 2004; Valente et al., 2024; Salman et al., 2022). Its perivascular localization further supports endothelial tight junction stability and BBB ultrastructure (e.g., astrocytic endfeet abut microvessels, influencing endothelial morphology) (Wolburg et al., 2012; Mueller et al., 2023). The BBB is anatomically adjacent to the perivascular spaces engaged by glymphatic flow. Therefore, AQP4 at the astrocyte provides a molecular interface for exchange, between vascular and perivascular/glymphatic compartments. Alterations in AQP4 expression, or loss of perivascular polarity can disturb endfoot–vascular coupling, degrade tight-junction integrity, increase BBB permeability, and exacerbate vasogenic edema in injury or disease states (Mueller et al., 2023; Jeon et al., 2021; Zhou et al., 2008). In GBM, loss of AQP4 polarity and disruption of its perivascular organization correlate with increased blood–brain barrier permeability, as demonstrated by elevated sodium fluorescein leakage and higher MRI-derived edema indices in patient specimens (Valente et al., 2022; Solar et al., 2022; Abbrescia et al., 2024). This breakdown allows plasma proteins and water to enter the brain parenchyma, resulting in vasogenic edema, and perivascular disorganization (Wolburg et al., 2012) that in turn impairs glymphatic clearance (Valente et al., 2022; Gao et al., 2024)—two coupled yet separable processes (Warth et al., 2007; Warth et al., 2004; Valente et al., 2024; Wolburg et al., 2012; Noell et al., 2012).

**Figure 3:**
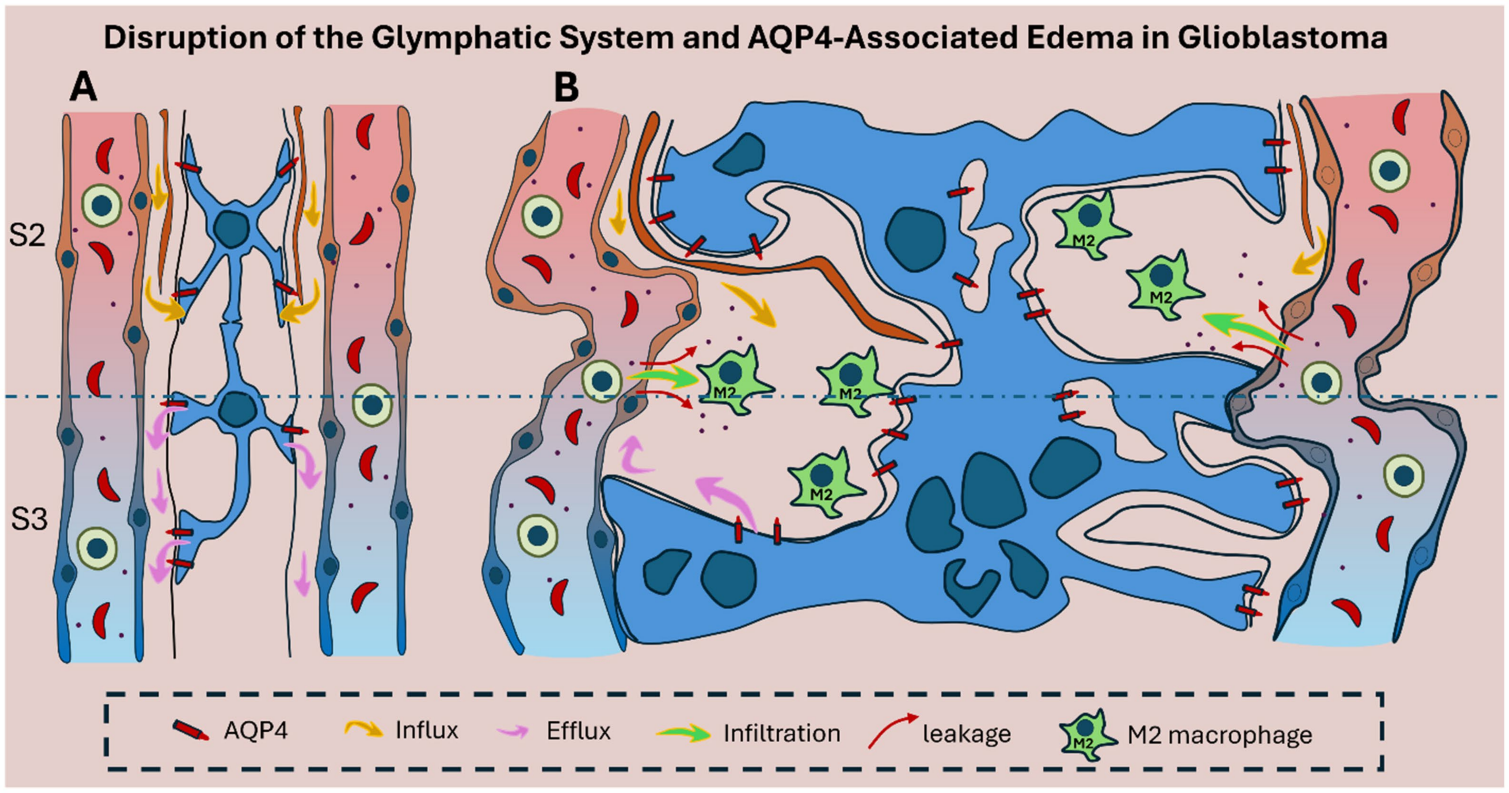
Schematic representation of glymphatic system disruption in glioblastoma. **(A)** In healthy brain tissue, the glymphatic system supports directional CSF–ISF exchange through intact Virchow–Robin spaces in zones S2 and S3. Polarized AQP4 is concentrated at astrocytic endfeet, enabling efficient perivascular fluid movement and metabolic waste clearance. **(B)** In glioblastoma, glymphatic architecture is disrupted by glial proliferation, vascular alteration, and tumor-induced structural disorganization. AQP4 becomes mislocalized, reducing directional flow and promoting peritumoral edema. CSF influx (yellow arrows) and impaired efflux (pink arrows) cause fluid accumulation, while increased vascular permeability allows leakage of immune cells (green) and blood-derived products (red arrows), reflecting local BBB disruption that further amplifies edema and inflammation. In addition, M2-polarized macrophages sustain a tumor-permissive microenvironment.

Mechanistically, the mislocalization of AQP4 is associated with the degradation of anchoring proteins such as agrin and components of the dystrophin–dystroglycan complex, which are responsible for tethering AQP4 to the astrocytic endfoot membrane (Warth et al., 2004; Wolburg et al., 2012; Noell et al., 2012). The detachment of AQP4 from these complexes not only disrupts water channel function but also undermines the scaffold supporting the BBB (Figure 3).

Understanding the link between AQP4 localization and BBB stability could affect therapeutic approaches aimed at restoring vascular integrity. By reestablishing the polarized expression of AQP4 and preserving the dystrophin–dystroglycan complex, it may be possible to reinforce BBB function and attenuate tumor-associated edema and invasion.

## Glymphatic dysfunction, AQP4 dysregulation, and peritumoral edema in glioblastoma

Anatomical and functional disturbances in the GS are increasingly recognized as contributors to the pathophysiology of glioblastoma. In particular, tumor-induced compression, glial proliferation, and vascular disorganization disrupt directional perivascular flow and glymphatic clearance, changes that are most pronounced in the CSF–ISF exchange (S2) and perivenous efflux (S3) regions, with reduced meningeal outflow in the newly defined S4 zone as reported in glioma (Rasmussen et al., 2018; Gao et al., 2024; Scalia et al., 2024) (Figure 3).

**Figure 4:**
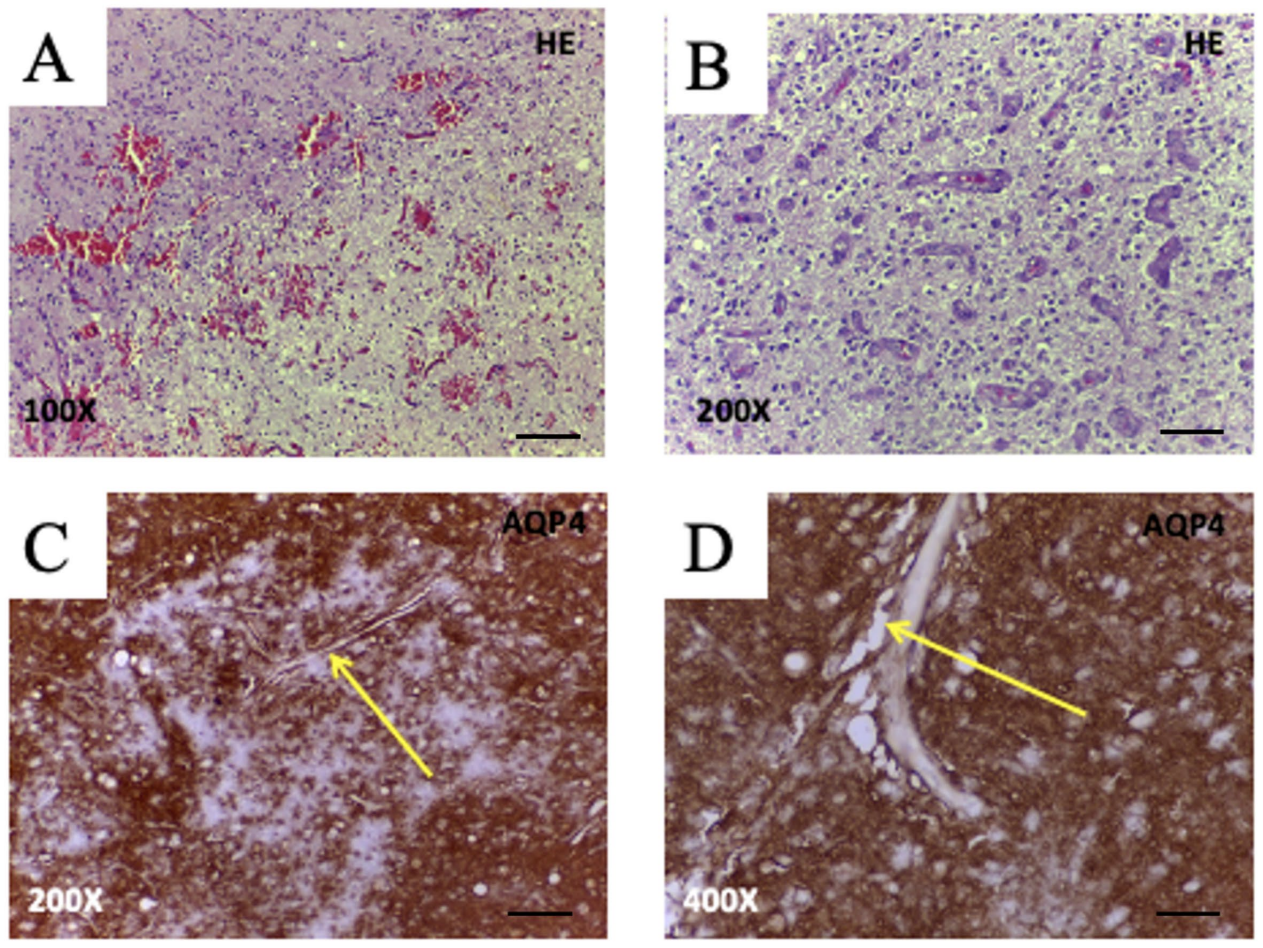
Histopathological features and AQP4 expression in glioblastoma (WHO Grade IV). **(A,B)** Hematoxylin and eosin (H&E) staining of glioblastoma tissue reveals hallmark features, including moderate hypercellularity and geographic necrosis (**A**, 100X; bar 200 μm), and prominent microvascular proliferation with glomeruloid-like vessels (**B**, 200X; bar 100 μm). **(C,D)** Immunohistochemistry for AQP4 shows strong perivascular labeling at astrocytic endfeet (arrows), highlighting pathological vessels surrounded by AQP4 + glial processes (**C**, 200X; bar 100 μm) and the presence of irregular, dilated perivascular spaces (**D**, 400X; bar 50 μm). These findings support AQP4’s role in tumor-associated vascular remodeling, glial proliferation, and potentially impaired glymphatic clearance in glioblastoma. Images from unpublished clinical case from our group.

Advanced MRI techniques have revealed significantly reduced glymphatic activity in GBM patients, correlating with interstitial fluid retention and peritumoral edema (Mueller et al., 2023). These disruptions are closely associated with changes in AQP4 expression and distribution. In healthy brain tissue, AQP4 is highly polarized at astrocytic end-feet lining the perivascular spaces, facilitating the exchange of CSF and ISF, as well as the clearance of metabolic waste (McCoy et al., 2010; Mou et al., 2010). In GBM, however, AQP4 becomes disorganized—often redistributed throughout the astrocytic membrane or even overexpressed in tumor cells—thereby compromising tumor-associated perivascular fluid transport (Noell et al., 2012; Mou et al., 2010) as illustrated schematically in Figure 3 and histologically in Figure 4.

Histological analyses have shown that AQP4 is frequently upregulated around aberrant tumor vasculature and accumulates near dilated or disorganized perivascular spaces (Warth et al., 2007; Gao et al., 2024; Noell et al., 2012; Scalia et al., 2024) (Figure 4). Such alterations not only disrupt local water homeostasis but also impair perivascular fluid transport, contributing to tissue swelling and elevated intracranial pressure. Consistent with this, Scalia et al. (2024) reported that GBM-associated glymphatic dysfunction involves reduced meningeal lymphatic drainage and loss of directional fluid clearance. In glioblastoma, AQP4 is often mislocalized or overexpressed within tumor cells, resulting in loss of perivascular polarization and impaired coordination of perivascular flow. Experimental models demonstrate reduced glymphatic influx and clearance in tumor-bearing hemispheres (Kaur et al., 2023) while diffusion MRI studies in patients reveal diminished perivascular flow indices (lower DTI-ALPS) in tumor-affected regions (Liang et al., 2025). Given that polarized AQP4 expression at astrocytic endfeet is essential for CSF–interstitial fluid exchange (Simon et al., 2022; Peng et al., 2023) its disruption in GBM likely compromises glymphatic transport not only within the tumor core but also in surrounding brain tissue, thereby promoting peritumoral fluid accumulation and impaired metabolic waste clearance.

The edema is a hallmark of glioblastoma and contributes substantially to the clinical symptoms and poor prognosis associated with the disease. Traditionally considered a passive consequence of tumor growth, peritumoral edema is now recognized as an active process involving glymphatic dysfunction and astrocytic activation. The breakdown of glymphatic flow due to tumor expansion and vascular alteration results in the accumulation of interstitial and cerebrospinal fluid in the peritumoral region (Ma et al., 2019; Scalia et al., 2024; Lan et al., 2023; Siri et al., 2024).

AQP4 plays a central role in this process. Elevated expression and mislocalization of AQP4 around tumor boundaries are strongly correlated with the extent and severity of peritumoral edema (Gao et al., 2024; Noell et al., 2012; Mou et al., 2010). Rather than facilitating efficient fluid clearance, disorganized AQP4 expression disrupts osmotic gradients and contributes to aberrant water influx into the extracellular space, thereby worsening local swelling.

Therapeutically, strategies aimed at modulating AQP4 distribution—either through pharmacologic means or gene-targeted approaches—may offer a way to reduce edema and improve clinical outcomes. Additionally, restoring the integrity of the glymphatic system may help re-establish fluid balance and relieve peritumoral mass effect. As such, understanding the bidirectional relationship between AQP4 function and glymphatic flow is essential for the development of targeted interventions to mitigate edema in GBM.

## AQP4 in immune modulation and the tumor microenvironment

**Figure 5:**
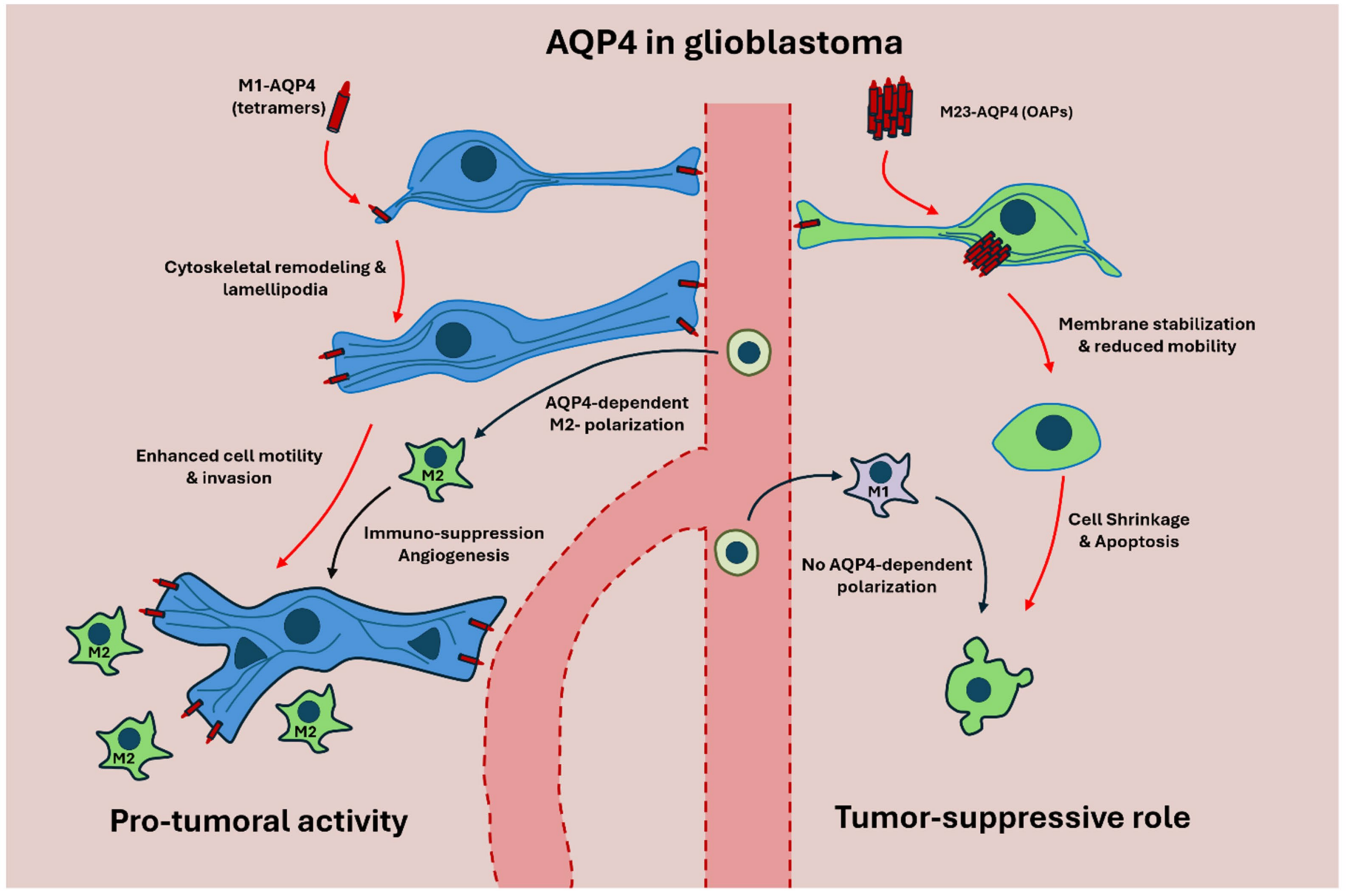
Schematic representation of M1-AQP4 and M23-AQP4 isoforms in glioblastoma (GBM). On the left, M1-AQP4, which forms non-OAP tetramers, predominates in GBM and drives cytoskeletal remodeling and lamellipodia formation, thereby enhancing glioma cell motility, invasion, and extracellular-matrix remodeling. This M1-dominated state is further associated with polarization of tumor-associated macrophages (TAMs) toward the immunosuppressive M2 phenotype, promoting angiogenesis, immune evasion, and tumor progression. On the right, M23-AQP4 assembles into OAPs that stabilize astrocytic membranes and maintain polarized perivascular water flux under physiological conditions. In this configuration, AQP4 supports glymphatic flow, limits cell migration, and favors apoptosis, reflecting a tumor-suppressive and homeostatic profile. Together, these isoform-specific mechanisms illustrate how the M1/M23 balance governs both normal astrocytic physiology and the malignant as well as immunological behavior of GBM.

Beyond water transport, AQP4 contributes to cellular dynamics and to the immune architecture of glioblastoma; higher AQP4 expression is associated with M2-like tumor-associated macrophage (TAM) enrichment and immune evasion (Lan et al., 2022; Mou et al., 2010). One of its critical functions lies in driving TAM polarization toward the M2 phenotype—a state characterized by immune suppression, angiogenesis, and tissue remodeling (Lan et al., 2022; Wang et al., 2023) (Figure 5). M2-polarized TAMs secrete anti-inflammatory cytokines such as IL-10 and TGF-*β*, support extracellular matrix reorganization, and suppress antigen presentation, thereby contributing to immune evasion and tumor growth. High AQP4 expression in glioma tissues has been associated with a higher prevalence of M2-like TAMs, linking AQP4 to an immunosuppressive tumor niche (Pisani et al., 2021; Lan et al., 2022; Wang et al., 2023; Seifert et al., 2015; Simone et al., 2022; Du et al., 2020).

Recent single-cell RNA sequencing studies further support this relationship by demonstrating shifts in macrophage polarization states in AQP4-high gliomas (Wang et al., 2023). Moreover, AQP4-containing extracellular vesicles have been shown to influence the phenotype of surrounding immune cells and contribute to the remodeling of the tumor milieu (Simone et al., 2022).

Taken together, these findings highlight the immunomodulatory potential of AQP4, underscoring its role beyond its function as a water channel. By orchestrating both glioma cell motility and immune evasion, AQP4 emerges as a dual-threat molecule within the GBM microenvironment. Therapies aimed at modulating AQP4 expression or localization may not only impede tumor spread but also recalibrate the immune landscape toward an anti-tumoral state.

## AQP4 as a therapeutic target in glioblastoma

One of the most challenging clinical features of glioblastoma is its resistance to therapies, including radiation and chemotherapy. AQP4 has been implicated in maintaining therapy-resistant niches, particularly in regions of hypoxia and low perfusion where slow-cycling, stem-like glioma cells persist (Jia et al., 2023; MacLeod et al., 2019). These microenvironments exhibit altered water-efflux kinetics, suggesting that AQP4 may support cellular survival under cytotoxic stress by preserving ionic balance in hypo-perfused tumor zones, where drug diffusion is limited.

AQP4-enriched astrocytic domains in gliomas often co-localize with regions exhibiting poor therapeutic penetration, hinting at a protective spatial arrangement that shelters vulnerable glioma subpopulations. In such zones, AQP4’s role in maintaining cell polarity and local fluid dynamics may buffer against treatment-induced apoptosis, enabling tumor cell regrowth (Ding et al., 2011).

Beyond its role in water transport, AQP4 contributes to glioma cell migration and invasion through dynamic cytoskeletal remodeling, membrane plasticity, and interactions with ion channels (Ding et al., 2010; Varricchio et al., 2021). Upstream metabolic signaling via the IDO1/TDO–kynurenine (Kyn) pathway activates the AhR, which upregulates AQP4 and biases isoform balance toward invasive phenotypes; AQP4 then acts as a downstream effector that promotes motility, immune tolerance, and survival under therapeutic stress (Du et al., 2020; Varricchio and Yool, 2023).

Pharmacologic inhibition of AQP4—alone or in combination with ion channel modulators—has shown promise in preclinical models by reducing glioma cell motility, sensitizing tumors to standard therapies, and suppressing recurrence (Varricchio et al., 2021; Varricchio and Yool, 2023). For example, TGN-020, a selective AQP4 inhibitor, has demonstrated efficacy in reducing cerebral edema and glioma cell migration in animal models, while AER-271, a more recent compound, has shown similar effects with improved pharmacokinetics and CNS penetration (Behnam et al., 2022). However, both agents face challenges in clinical translation due to limited blood–brain barrier (BBB) permeability and potential off-target effects. To overcome these limitations, novel delivery strategies such as nanoparticle-based platforms and BBB-penetrating antibody conjugates are being explored. Simultaneous inhibition of AQP4 and co-expressed ion channels has shown synergistic effects in reducing glioma cell viability (MacLeod et al., 2019). Targeting upstream regulators like the Kyn–AhR–AQP4 axis also offers a promising approach to disrupt glioma proliferation and immune evasion (Du et al., 2020).

Altogether, AQP4 represents a mechanistically grounded and multifaceted therapeutic target in GBM. Future efforts should prioritize the development of BBB-permeable inhibitors, identification of AQP4-driven patient subgroups, and clinical evaluation of AQP4-directed interventions to improve long-term outcomes.

## AQP4 isoforms and functional roles in gliomas

AQP4 exists in multiple isoforms, primarily M1-AQP4 and M23-AQP4, which differ in their N-terminal sequences and in their ability to form supramolecular structures (Pisani et al., 2021; Smith et al., 2014; de Bellis et al., 2021). These isoforms play divergent roles in glioma biology, particularly influencing cellular motility, invasiveness, and the structural integrity of astrocytic networks.

M1-AQP4, typically forming tetramers, is associated with increased astrocyte migration and invasive capacity in high-grade gliomas. Its expression correlates with poorer prognosis, increased recurrence rates, and greater tumor spread (Sun et al., 2020; Engelhorn et al., 2009). M1-AQP4 enhances the formation of lamellipodia—actin-rich membrane protrusions—facilitating dynamic cytoskeletal remodeling required for glioma cell motility (Smith et al., 2014; Lan et al., 2017) (Figure 5).

In contrast, M23-AQP4 forms orthogonal arrays of particles (OAPs) that stabilize the astrocyte plasma membrane and restrict cell motility. These arrays support a more polarized cellular phenotype, associated with membrane rigidity and induction of apoptosis (Amiry-Moghaddam, 2019; Simone et al., 2019; de Bellis et al., 2021; McCoy and Sontheimer, 2007; Hiroaki et al., 2006). Thus, the relative abundance and distribution of AQP4 isoforms can influence the invasive phenotype of glioma cells (Figure 5).

Molecular regulators of AQP4 isoform expression have also been identified. Thus, The Kyn–AhR axis also influences AQP4 expression by favoring the upregulation of the M1-AQP4 isoform, thus reinforcing the pro-invasive and therapy-resistant phenotype of glioma cells (Du et al., 2020). This metabolic-immunologic axis represents a key interface between tumor metabolism and membrane protein regulation. Moreover, non-coding RNAs such as lncRNA LINC00461 and miR-216a modulate AQP4 expression, further linking gene regulation to tumor behavior (Behnam et al., 2022).

These findings emphasize that AQP4 isoform switching is not merely a structural adaptation but may actively drive glioma progression. Understanding and manipulating this isoform balance could represent a novel therapeutic strategy in GBM.

## Clinical intersection of AQP4 function: glioblastoma and neuromyelitis optica spectrum disorders (NMOSD)

The therapeutic significance of AQP4 has prompted the investigation of the intersection between glioblastoma and NMOSD, an autoimmune condition characterized by the presence of pathogenic anti-AQP4 antibodies (Castañeyra-Ruiz et al., 2014). Although chronic AQP4 inhibition in NMOSD may hypothetically decrease the risk of glioma development, there is currently insufficient evidence to substantiate this hypothesis.

Numerous case reports have underscored the diagnostic challenges and occasional clinical overlap between NMOSD and gliomas, primarily due to the risk of misdiagnosis in which often NMOSD is erroneously identified as brain tumors located in regions where AQP4 is abundantly expressed (Park and Hwang, 2021; Song et al., 2020; Tomari et al., 2024; Natsis et al., 2021).

A particularly intriguing case highlighted the coexistence of NMOSD and glioblastoma, where the brain tumor emerged while the patient was undergoing AQP4 immunosuppressive treatment for NMOSD. In this situation, the immunosuppression may have prevented the autoimmune system from identifying and targeting the glioblastoma’s AQP4, facilitating its growth. Notably, upon partial withdrawal of immunosuppressive therapy, NMOSD re-emerged while the glioblastoma entered remission (Donovan et al., 2017). This paradoxical outcome suggests a potential immunological conflict between active NMOSD and glioblastoma progression, reinforcing the notion that AQP4-targeted autoimmunity could inherently oppose glioma advancement. Remarkably, Liao et al. (2010) demonstrated that serum from NMOSD patients, rich in anti-AQP4 antibodies, exhibited reactivity against human glioblastomas. While anti-AQP4 antibodies are widely considered pathogenic in NMOSD, some studies suggest they may represent an epiphenomenon rather than a direct driver of disease pathology (Schmetzer et al., 2021). Although this does not undermine the immunological relevance of AQP4, it introduces nuance into the role of recomanti-AQP4 antibodies in potential therapeutic implications for glioblastoma.

One of the most striking features of glioblastoma is its ability to spread through the perivascular spaces of Virchow–Robin, a phenomenon known as perivascular satellitosis or satellitism. This invasive behavior contributes to the tumor’s poor surgical treatability and is mediated by chemokines, such as stromal cell-derived factor 1 alpha (SDF-1α or CXCL12), which is expressed in subpial blood vessels (Wesseling et al., 2011). Glioblastoma cells express corresponding receptors, including CXCR4, which facilitates chemotactic migration (Wesseling et al., 2011; Esencay et al., 2013; Zagzag et al., 2008). Intriguingly, an inverse relationship has been reported between anti-AQP4 autoantibodies and CXCL12 levels in NMOSD patients, suggesting a potential immunological conflict between NMOSD and glioblastoma progression eritumoral astrocytic mislocalization of AQP4, together with tumor mass effect, compromises glymphatic function by distorting perivascular spaces and compressing CSF–ISF exchange zones (Kang et al., 2015). Moreover, homologous aquaporins, such as AQP3, are known to be upregulated at the migrating edge of cancer cells upon stimulation with CXCL12, further implicating aquaporins in chemokine-driven tumor invasion (Satooka and Hara-Chikuma, 2016).

Given the limited data available, these findings suggest an immunological incompatibility between active NMOSD and glioma progression that warrants further exploration. Systematic reviews and large multicenter patient registries could provide crucial insights into whether NMOSD offers any protective effect against glioma development and whether AQP4-targeted immunity could be therapeutically exploited. These observations should be interpreted with caution, yet they remain valuable for generating new hypotheses.

## Conclusions and future directions

This review highlights the central role of aquaporin-4 (AQP4) in glioblastoma pathophysiology, extending beyond its canonical function in water transport to include tumor-cell migration, immune modulation, blood–brain barrier (BBB) disruption, and therapeutic resistance. AQP4 dysfunction—particularly its isoform-specific expression and subcellular mislocalization—emerges as a converging mechanism linking glymphatic system disruption with glioma progression.

We propose a refined anatomical framework for the glymphatic system composed of four segments, each with distinct structural and functional roles in cerebrospinal fluid (CSF)–interstitial fluid (ISF) exchange. This segmentation offers new insights into how specific anatomical zones may be selectively compromised in GBM, resulting in impaired waste clearance, edema formation, and immune evasion.

Therapeutically, AQP4 represents a promising but complex target. Pharmacological inhibitors, isoform-specific regulators, and novel delivery systems (e.g., nanoparticle-conjugated agents) offer avenues for disrupting AQP4-mediated tumor support. However, aquaporins are traditionally considered “undruggable”; therefore, future strategies may focus on modulating their upstream regulatory pathways or selectively interfering with pore function. Moreover, the IDO1/TDO–Kyn–AHR–AQP4 signaling axis, together with AQP4’s interaction with tumor-associated immune components such as macrophages, warrant the exploration of combined therapeutic approaches.

The rare clinical intersection between glioblastoma and NMOSD suggests a potentially exploitable immunological relationship, though further data are required to substantiate therapeutic applications.

Future research should focus on (1) clarifying causal links between AQP4 dysregulation and glymphatic failure in gliomas, (2) identifying biomarkers of AQP4 activity and localization for patient stratification, and (3) developing BBB-penetrant, isoform-selective interventions. Such approaches may improve diagnosis, therapeutic targeting, and clinical outcomes in glioblastoma and related central nervous system tumors.
